# Progress in the research and development of oncolytic virus therapies

**DOI:** 10.3389/fphar.2026.1751206

**Published:** 2026-02-19

**Authors:** Yiran Li, Xi Qin, Chenggang Liang, Lan Wang

**Affiliations:** 1 National Institutes for Food and Drug Control, Beijing, China; 2 State Key Laboratory of Drug Regulatory Science, National Institutes for Food and Drug Control, Beijing, China

**Keywords:** clinical research, combination therapy strategy, genetic engineering modification, oncolytic virus, quality control

## Abstract

Oncolytic viruses (OVs) are a class of viral preparations with selective replication capability in tumor cells and the ability to activate systemic anti-tumor immunity. They have emerged as an important breakthrough in cancer treatment following chemotherapy, targeted therapy, and immune checkpoint inhibitors. This article systematically reviews the developmental trajectory of OVs from the accidental discovery of wild strains to their genetic engineering-based modification and optimization, and subsequently to accelerated clinical translation. It primarily highlights key advances in viral backbone design, immune regulatory gene insertion, and combination therapy strategies. Currently, several OV-based therapeutics have been approved for clinical use worldwide for the treatment of various solid tumors, including melanoma, glioblastoma, and head and neck cancers, demonstrating their extensive potential for broader indication coverage as evidenced by ongoing clinical trials. Although OVs possess unique advantages in their ability to remodel the tumor microenvironment and elicit both local and systemic anti-tumor effects, their clinical application still faces challenges such as limited monotherapy efficacy, barriers to systemic delivery, a lack of precision biomarkers, and issues in large-scale manufacturing and quality control. Looking ahead, by drawing on cutting-edge technologies such as CRISPR-based gene editing, reverse genetics, advanced delivery systems, and multimodal combination therapy, OVs are expected to achieve greater precision and personalization in cancer treatment, thereby promoting their wider application in the management of solid tumors.

## 1 Introduction

Throughout the evolution of cancer therapy, traditional chemotherapy has been defined by strong cytotoxic effects but poor selectivity, whereas targeted therapies offer higher precision but are prone to drug resistance. Immunotherapies can activate the immune system but exhibit limited response rates. Against this backdrop, oncolytic viruses (OVs) have emerged as a promising next-generation therapeutic strategy, owing to their dual properties of tumor selectivity and immune activation. OVs can specifically recognize and replicate within tumor cells, inducing tumor cell death through direct oncolytic effects. The tumor-associated antigens (TAAs) released during tumor cell lysis subsequently stimulate systemic anti-tumor immunity, thereby contributing to the elimination of residual and metastatic lesions (Kaufman et al., 2016). Therefore, OVs, as dual-functional viral preparations, offer broad prospects for clinical translation. The structural characteristics of OVs, such as the presence or absence of an envelope and the degree of genetic modification, directly influence infection specificity, replication control, immunogenicity, and transgene-loading capacity (Fukuhara et al., 2016). Preclinical and clinical evidence has shown that OVs offer significant advantages in terms of safety, efficacy, diversity of administration routes, drug resistance management, expansion of indications, and sustained response potential (Kaufman et al., 2016). They have been used to treat multiple tumor types and disease stages, including metastatic and refractory solid tumors, and they can significantly prolong the overall survival (OS) of patients (Phelps et al., 2018). For patients with advanced disease, OV treatment can not only prolong OS, but also may lead to complete (CR) or partial responses (PR) (Boorjian et al., 2022).

At present, OV-related clinical trials have been conducted throughout the globe for the treatment of solid tumors such as melanoma, bladder cancer, pancreatic cancer, ovarian cancer, glioblastoma, head and neck tumors, and sarcomas, highlighting their potential for cross-tumor application (Fukuhara et al., 2016). Public data show that six OV products have been approved globally, signifying a major transition from conceptual validation to clinical availability (Macedo et al., 2020). Moreover, a search on *clinicaltrials.gov* indicates over 200 ongoing clinical trials of oncolytic viruses alone or in combination with other therapies, reflecting the rapid expansion of the OV research pipeline (Harrington et al., 2019). Nevertheless, the clinical implementation of OV-based therapies still faces multiple challenges, such as limited monotherapy efficacy, drug resistance, barriers to systemic delivery, the need for precise indication screening, and large-scale production challenges (Shalhout et al., 2023). This review summarizes the evolution of OVs from basic research to clinical translation, with particular focus on process optimization and quality control requirements, with the aim of providing a reference for subsequent research and development, registration, and standardized clinical applications.

## 2 The selective replication and targeting mechanism of oncolytic viruses

Normal cells possess a complete innate antiviral defense system, with the core being the type I interferon signaling pathway and its downstream effectors (such as protein kinase R, PKR). When a virus invades, this pathway is rapidly activated, inducing the cell to enter an antiviral state, thereby effectively inhibiting viral replication. However, in many malignant tumors, this defense network often exhibits functional defects or expression downregulation, resulting in a significant reduction in the ability of tumor cells to resist viral infection. This “immune deficiency” state makes tumor cells ideal sites for viral replication, forming the basis of the natural tendency of oncolytic viruses (Kaufman et al., 2015). Based on this, genetic engineering strategies can be precisely implemented. For example, by deleting the neurovirulence gene ICP34.5 in the herpes simplex virus (HSV) that inhibits the host PKR pathway, the modified virus (such as T-VEC and G47Δ) replicates with limited ability in healthy cells with normal PKR function, but can effectively replicate and spread in tumor cells with dysregulated PKR signaling, thereby achieving selective amplification in tumor tissues (Todo et al., 2022a).

To further enhance the specificity and safety of the targeted approach, researchers have developed various molecular strategies for actively regulating the fate of the virus. These strategies can be implemented at the transcriptional level, where the key genes necessary for virus replication are placed under the control of tumor-specific or related promoters (Nettelbeck, 2008); they can also be regulated at a post-transcriptional level, by embedding specific complementary target sequences of microRNAs (miRNAs) in the viral genome, to achieve precise regulation of the expression of exogenous genes (Ylösmäki and Cerullo, 2020); and they can also modify the virus in a directional manner, by engineering the viral capsid or envelope proteins, which can enhance its affinity for tumor-expressed receptors, and even redirect its directionality to a completely new tumor-specific antigen, thereby achieving precise localization at the initial stage of infection (Waehler et al., 2007).

## 3 Progress in the study of oncolytic virus therapies

Based on their genetic composition, OVs can be classified into DNA viruses (e.g., adenovirus, vaccinia virus [VV], herpes simplex virus [HSV]) and RNA viruses (e.g., Newcastle disease virus [NDV], coxsackievirus, reovirus). DNA viruses possess larger and more stable genomes, making them more conducive to the insertion of exogenous genes (Macedo et al., 2020). In contrast, RNA viruses exhibit robust replication, high immunogenicity, and small particle size, making them conducive to systemic diffusion (Prestwich et al., 2009) (Table 1).

**TABLE 1:** The main classes of oncolytic viruses (OVs).

Characteristics	Adenovirus (Moss et al., 2013)	Vaccinia virus (Knipe and Howley, 2021)	Herpes virus (Ganar et al., 2014)	Newcastle disease virus (−ssRNA) (Danthi et al., 2010)	Reovirus (dsRNA) (Coyne and Bergelson, 2006)	Coxsackievirus (+ssRNA) (Dock, 1904)
Genetic material	Double-stranded DNA	Double-stranded DNA	Double-stranded DNA	Single-stranded RNA	Double-stranded RNA	Single-stranded RNA
Genome size	36–38 kbp	192 kbp	152 kbp	15.2 kb	23.5 kbp	7.4 kb
Virus size	Diameter approximately 90–100 nm	Polymorphic, brick-shaped, approximately 350 × 270 nm	150–200 nm	100–500 nm, polymorphic	70–80 nm	30 nm
Envelope presence	No	Yes	Yes	Yes	No	No
Known or speculated cell receptors	Coxsackievirus-adenovirus receptor (CAR), CD46, heparan sulfate proteoglycan, integrin αvβ3/αvβ5	Heparin sulfate proteoglycans, laminin receptors, chemokine receptors	Heparin sulfate proteoglycans mediate initial binding; Nectin-1, HVEM., mediated entry	Sialic acid	Sialic acid, JAM-A	ICAM-1, DAF

Initially, researchers believed that OVs exerted their anti-cancer effects mainly through direct viral oncolysis, with their efficacy largely depending on how OVs effectively propagate and spread through the tumor microenvironment to infect and kill cancer cells (Wang et al., 2022). However, recently, an increasing number of studies have shown that the innate and adaptive immune systems play significant roles in the powerful, long-lasting anti-cancer effects of OVs. OV infection has been shown to induce inflammatory reprogramming within the tumor microenvironment (Appleton et al., 2021), including altered immune cell infiltration, matrix disruption, interferon (IFN)-γ expression, and the upregulation of tbe PD-1/PD-L1 axis (Ribas et al., 2017).

### 3.1 Phases of oncolytic virus development

**FIGURE 1:**
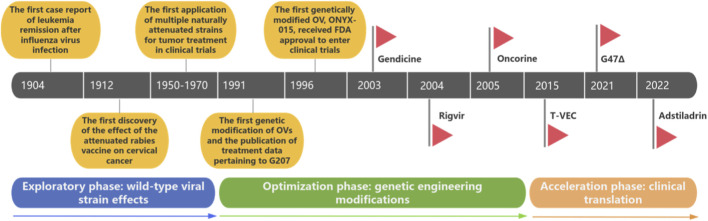
The development stages of OVs (De Pace, 1912; Kelly and Russell, 2007; Huebner et al., 1957; Hunter-Craig et al., 1970; Liu et al., 2007; Martuza et al., 1991; Bischoff et al., 1996; Ganly et al., 2000; Kirn, 2001; Liu et al., 2003; Colbert et al., 2025; Guo et al., 2023; Zhang et al., 2018; Khuri et al., 2000; Kohlhapp and Kaufman, 2016; Phelps et al., 2018).

The development of OVs can be divided into three phases: (1) the exploratory phase involving wild-type viral strains, (2) the optimization phase characterized by genetic engineering modifications, and (3) the acceleration phase focusing on clinical translation (Figure 1). At the beginning of the 20th century, cases of viral infection accompanied by spontaneous tumor regression were first reported. In the 1950s and 1970s, clinical trials of attenuated wild-type strains were terminated due to insufficient safety and targeting. In the 1990s,The precise knockout of virulence genes and the insertion of therapeutic genes represented a significant leap forward in OV safety and efficacy. In the 21st century, with the successive approvals of products such as T-VEC and H101, OVs officially entered the stage of clinical application. The following sections review the development of OV drugs from natural wild OVs to genetically modified OV platforms.

#### 3.1.1 The exploratory phase of wild-type OV strains

In 1904, Dock reported a 42-year-old female patient with chronic myeloid leukemia who had a significant decrease in white blood cell count after influenza virus infection, first suggesting a potential association between viral infection and reduced tumor burden (De Pace, 1912). In 1912, De Pace observed that a cervical cancer patient who had not undergone surgery showed tumor regression after multiple doses of rabies vaccine, which further strengthened the hypothesized virus-tumor interaction and is now considered to represent the starting point for more intensive research exploring viral tumor therapy (Kelly and Russell, 2007; Huebner et al., 1957). In the 1950s, Huebner et al. applied natural adenovirus to treat 30 patients with cervical cancer, and found that infectious virus could be isolated from about 66% of tumor specimens. More than 50% cases showed local remission, but later disease progression occurred in all cases (Hunter-Craig et al., 1970). In the 1970s, Hunter-Craig et al. studied the effect of intratumoral injection of natural vaccinia virus (VV) in the treatment of metastatic melanoma. They found that 57% of patients exhibited objective responses at the injection site, among whom 6 patients presented with disease-free survival for 2 years, suggesting that VV may have induced sustainable treatment outcomes (Liu et al., 2007). Subsequently, researchers also examined the therapeutic potential of other naturally occurring viruses, including HSV, West Nile virus (WNV), mumps virus (MV), and NDV, in various tumor types (Martuza et al., 1991).

#### 3.1.2 The genetic engineering-driven optimization phase

In the mid-20th century, the natural viruses used for anti-tumor treatment were mainly pathogenic viruses, posing significant safety risks and offering limited therapeutic effects. In the 1990s, with the development of genetic engineering technology, researchers had the opportunity to further modify and optimize these naturally occurring viruses to improve their safety, efficacy, and targeting, providing a solid basis for the clinical application of OV therapy.At the same time, researchers have also begun to pay attention to the unexpected mutations that the virus may undergo during the genetic modification process, especially those that could cause the virus to target normal cells rather than tumor cells. These are the key risks that must be strictly avoided in the development of oncolytic virus therapy. However, the successful application of oncolytic virus therapy is not solely dependent on the optimization of the virus itself. In clinical translation, the interaction between the virus and the target cells is the key foundation for the therapeutic effect to take effect. However, the human immune system constitutes a major barrier. The immune system may recognize and eliminate exogenous viruses before the virus reaches or infiltrates the tumor tissue. Moreover, the pre-existing immunity in the patient’s body due to previous infections (such as neutralizing antibodies) can further accelerate the neutralization and clearance of the virus, thereby significantly weakening the replication and oncolytic effect of OV in the tumor site (Mastrangelo et al., 1999). This immune clearance mechanism is the core factor limiting the efficacy of systemic administration of OV and has also become an important research direction for promoting subsequent virus engineering modifications (such as inserting immune regulatory genes) and optimizing the administration strategy (Jhawar et al., 2017). In 1991, the Martuza team first reported the use of genetically modified HSV G207 to treat glioma, knocking out the ICP34.5 neurotoxicity gene and inactivating the ICP6 gene, thereby laying the foundation for OV gene editing (Bischoff et al., 1996). In 1996, the first molecular engineering-based OV, ONYX-015, was approved by the US Food and Drug Administration (FDA) for clinical trials. This virus was an adenovirus with an E1B-55kD gene deletion and was designed to selectively replicate in tumor cells exhibiting p53 dysfunction (Ganly et al., 2000). The subsequent phase I clinical trials confirmed the safety and feasibility of ONYX-015 for treating head and neck cancer via intratumoral injection (Kirn, 2001). This approval marked the official entry of OVs into clinical trials, beginning a new chapter in the research and application of OV therapies (Liu et al., 2003).

#### 3.1.3 The phase of accelerated clinical translation

To further enhance the anticancer activity of OVs, researchers have sought to improve their anti-tumor effects by introducing a range of exogenous genes. For example, T-VEC (talimogene laherparepvec), a modified HSV strain developed in the early 2000s, was developed by knocking out two copies of ICP34.5 (a neurotoxic factor) to enhance its tumor selectivity while introducing the GM-CSF (granulocyte-macrophage colony-stimulating factor) gene to enhance the immune response (Phelps et al., 2018). T-VEC was approved by the US FDA in 2015 for the treatment of metastatic melanoma, demonstrating both local and systemic anti-tumor effects (Todo et al., 2022a), marking the maturity of the OV technology. In 2021, Delytact (G47Δ), a type I oncolytic HSV developed by Japan’s Daiichi Sankyo Corporation, was also approved for marketing to treat malignant glioblastoma. The successful launch of this product further extended the clinical scope of OV therapy and demonstrated its potential in the treatment of refractory tumors (Colbert et al., 2025). In 2022, Adstiladrin from Ferring Pharmaceuticals also received FDA approval for marketing, making it the first and, to date, the only intravesical gene therapy approved for adult patients with non-muscle-invasive bladder cancer (NMIBC) but without response to standard treatment (Guo et al., 2023). This breakthrough not only provides new treatment options for bladder cancer patients but also marks another important application of OV therapy in the gene therapy field.

## 4 The marketed varieties and clinical research of oncolytic virus drugs

OVs can accurately identify and selectively replicate within tumor cells, effectively destroying tumor cells while maintaining safety by sparing surrounding normal tissues. Compared with traditional therapies, OVs offer significant advantages in terms of safety, efficacy, administration methods, drug resistance management, applicable diseases, and long-term benefits. They can potentially be used to target multiple types of tumors and disease stages, including metastatic or refractory cancers, thereby significantly prolonging patient OS. Clinical trials of OVs have been conducted for many solid tumors including melanoma, bladder cancer, pancreatic cancer, ovarian cancer, glioblastoma, head and neck tumors, and sarcoma, demonstrating broad application prospects (Peng, 2005). Table 2 provides a summary of representative OV types and relevant findings pertaining to their use in the management of major diseases.

**TABLE 2:** Oncolytic virus products available on the global market.

Product	Company	Country	Virus type	Indication	Approved year^a^
Gendicine	Sibiono	China	Recombinant Ad-p53 adenovirus	Head and neck squamous cell carcinoma	2003
Rigvir	Sia Latima	Latvia	Enterovirus ECHO-7	Melanoma	2004
Oncorine	Shanghai Sunway Biotech	China	Type 5 adenovirus	Nasopharyngeal carcinoma	2005
Imlygic (T-VEC)	Amgen	The United States	Type I herpes simplex virus	Melanoma	2015
Delytact (G47Δ)	Daiichi Sankyo	Japan	Type I herpes simplex virus	Glioblastoma	2021
Adstiladrin	Ferring	The United States	Adenovirus	Bladder cancer	2022

^a^ The listed years represent the time when the product was first approved for marketing in any country around the world.

Zhang et al., 2018; Doniņa et al., 2015; Kohlhapp and Kaufman, 2016; U.S. Food and Drug Administration, 2015; Boorjian et al., 2021; Lee, 2023.

### 4.1 Representative OV varieties on the market

Although Gendicine, Adstiladrin and Rigvir are often classified under the “virus-based anti-tumor” category, due to their lack (or extremely low level) of intratumoral replication and spread capabilities, they should strictly be regarded as “non-replicative viral gene therapies”, and significantly differ from replicative OV agents such as Oncorine, T-VEC, and G47Δ in their mechanism of action.

#### 4.1.1 Gendicine

Recombinant human p53 adenovirus injection, also known as Gendicine (Jinyou Sheng/Endicine), is an innovative biotherapeutic product developed by SiBiono Gene Technology Co., Ltd., designed to restore the normal function of wild-type p53 (Sabapathy and Lane, 2018). According to the International Agency for Research on Cancer database (http://p53.iarc.fr), *TP53* is one of the most frequently mutated genes in human cancer (Ma et al., 2017). Gendicine is a replication-defective recombinant human adenoviral vector that expresses the p53 protein and can inhibit the uncontrolled division of cancer cells, leading to their apoptosis (Zhang et al., 2024). Replication-defective human adenovirus type 5 acts as a vector to introduce the Tp53 gene into target cells, while the normal p53 tumor suppressor gene starts to express protein after entering tumor cells, thereby regulating a series of biological processes such as cell cycle arrest, apoptosis, cell aging, cell differentiation, angiogenesis, cell migration, metabolism, and DNA repair (Xia et al., 2020). These effects make recombinant human p53 adenovirus injection a promising approach to cancer treatment. In 2003, China became the first country to approve Gendicine (Zhang et al., 2018). By 2013, nearly 30,000 people had received Gendicine treatment, presenting a CR rate of 30%–40%, a PR rate of 50%–60%, and a cumulative remission rate exceeding 90% after treatment (Hietanen et al., 2022). Gendicine has been shown to be safe and effective in the treatment of various malignant tumors in preclinical tests and clinical trials.

#### 4.1.2 Rigvir

Rigvir is an innovative OV therapy developed by Latima Ltd. (Latvia) for the local treatment of melanoma, cutaneous and subcutaneous melanoma metastases, and for preventing recurrence or metastasis following radical surgery. Rigvir employs a cell-adapted enterovirus derived from an Echovirus 7 (E7) isolate, which selectively replicates in tumor cells, directly lyses malignant tissue, and activates systemic anti-tumor immunity through the release of danger signals (Alberts et al., 2018). In clinical settings, Rigvir has shown good safety and efficacy, leading to significantly improved survival rates among melanoma patients. In addition, Rigvir has also shown significant therapeutic effects against various solid tumors including gastrointestinal tumors, pancreatic cancer, cholangiocarcinoma, and malignant sarcoma (Doniņa et al., 2015). The development of Rigvir can be traced back to the late 1950s, in laboratories and clinics in Riga, Latvia (Doniņa et al., 2015). In 2004, Rigvir became the first OV drug registered by the State Agency of Medicines of the Republic of Latvia (Khuri et al., 2000). The regulatory status of Rigvir varies across different regions, and several jurisdictions are currently engaged in ongoing discussions regarding the strength of its clinical evidence.

#### 4.1.3 Oncorine/H101

Oncorine, trade name Acrel, is an innovative OV gene therapy product developed by Shanghai Sunway Biotechnology Co., Ltd., which is used to treat advanced head and neck tumors that do not respond to conventional radiotherapy or radiotherapy combined with chemotherapy. Oncorine was obtained by deleting the E1B-55K and E3 gene fragments of Ad5. This modification enables it to selectively replicate in tumor cells harboring p53 gene deficiencies or abnormalities without significant cytotoxic effects in normal cells (Oncorine, 2018). Oncorine is primarily used in combination with chemotherapy for the treatment of advanced head and neck cancers refractory to standard therapies, as well as in advanced nasopharyngeal carcinoma. Clinical studies have also demonstrated potential efficacy in advanced bladder cancer (Ma et al., 2009). The predecessor of Oncorine was ONYX-015, developed by Onyx Pharmaceuticals, which is a modified AdV that was first developed in the 1990s but discontinued in the early 21st century. Subsequently, the exclusive licence to ONYX-015 was granted to Shanghai Sunway Biotechnology Co., Ltd. in China, which developed the similar Oncorine virus (Wu et al., 2018). In 2005, the China National Medical Products Administration approved the product for marketing (Kohlhapp and Kaufman, 2016).

#### 4.1.4 T-VEC

Imlygic (T-VEC) is an advanced OV gene therapy developed by Amgen for melanoma treatment, especially cases that have metastasized to the skin, soft tissues, or lymph nodes and are not amenable to surgical removal. Imlygic is an engineered HSV-1 strain harboring GM-CSF gene insertion together with the knockout of the ICP34.5 and ICP47 genes (Zhang et al., 2017). GM-CSF gene insertion promotes the recruitment of antigen-presenting cells (APCs), and the activation of APCs enhances the presentation of tumor antigens to tumor-specific T cells, thereby further enhancing anti-tumor immunity (Liu et al., 2003). The knockout of the ICP34.5 gene enhances infection and replication activity within tumors, improves safety, and reduces the natural neurotoxicity of the virus (Hawkins et al., 2002). The knockout of the ICP47 gene allows the earlier and higher expression of the HSV unique short 11 (US11) gene, thereby increasing selectivity towards tumor cells (Hu et al., 2006). The development of T-VEC began in 1999 and was initiated by Dr. Robert Coffin’s research group at University College London. In 2006, T-VEC was first tested in a phase I clinical trial published by Hu et al. (Chesney et al., 2023). In October 2015, the US FDA approved T-VEC for the treatment of inoperable advanced melanoma, making it the first OV therapy and the first gene therapy approved in the United States (U.S. Food and Drug Administration, 2015). Ongoing clinical trials are investigating T-VEC in combination with immune checkpoint inhibitors (ICIs), such as ipilimumab and pembrolizumab, to assess its potential applications in a broader range of cancer types (Todo et al., 2001; Ribas et al., 2017).

#### 4.1.5 G47Δ

Delytact (G47Δ) was jointly developed by Daiichi Sankyo Corporation of Japan and the Institute of Medical Sciences of the University of Tokyo, and is an OV used for the treatment of glioma. G47Δ was created by adding a third mutation (α47 deletion) based on its predecessor virus G207. G207 is a second-generation, double-mutant recombinant oncolytic HSV lacking both copies of the ICP34.5 gene and containing an inactivated γ34.5 gene via insertion of the *E. coli* lacZ gene (Todo et al., 2022a). Inactivation of the ICP6 gene was also included. These modifications enable the virus to selectively infect and kill tumor cells while promoting antigen presentation and immune activity. In June 2021, G47Δ was conditionally approved by the Japanese Ministry of Health, Labour and Welfare for the treatment of malignant gliomas based on a 1-year survival rate of 84.2% in a single-arm phase II trial (Boorjian et al., 2021).

#### 4.1.6 Adstiladrin

Nadofaragene firedonovec (or Adstiladrin) was developed by Ferring Pharmaceuticals for the treatment of bladder cancer, which is the 10th most common cancer globally, with NMIBC accounting for about 80% of all cases. Adstiladrin is a gene therapy based on a nonreplicating viral DNA vector encoding IFN-α2b, leading to its upregulation in tumor cells, thereby triggering apoptosis. Other anti-tumor mechanisms include the induction of MHC-I expression and the promotion of anti-tumor T cell activity (Ferring Pharmaceuticals, 2024). In 2022, it was approved by the US FDA for marketing (Lee, 2023). After production process optimization, the product was fully launched across the United States in January 2024 (Ferring Pharmaceuticals, 2024; Lawler et al., 2017).

### 4.2 Clinical trial progress for OV drugs

According to the latest statistics from the ClinicalTrials database, as of September 2025, more than 200 relevant clinical trials have been registered worldwide. These clinical trials cover a wide range of virus types, tumor indications, and different clinical stages, demonstrating the diversity and wide applicability of OV therapy (Harrington et al., 2019). This field is thus entering an unprecedented period of accelerated development.

Among registered OV clinical trials, HSV is the most widely tested therapeutic agent, followed by adenovirus and smallpox virus. Other trials have also focused on measles virus, vesicular stomatitis virus, and reovirus (Clark et al., 2025). These different virus types reflect the diversity of research in the OV therapy field while also providing important references for future research.

Some OV trials have focused on the treatment of advanced malignant solid tumors. In July 2025, a phase II metastatic breast cancer trial confirmed that the combination of Pelareorep and paclitaxel was associated with increased toxicity, peripheral T cell clonal expansion, and increases in both objective response rate (ORR) and progression-free survival (PFS) (Yu et al., 2023). Pelaareorep is an unmodified non-pathogenic reovirus developed by Oncology Biotech, which has unique biological characteristics and can overcome the effects of neutralizing antibodies. Pelareorep selectively infects and destroys tumor cells by activating the host immune system, demonstrating broad prospects for application in the treatment of various solid tumors and hematological malignancies (Mori et al., 2019). Clinical studies have shown that the combination of the oncolytic VV GL-ONC1 and chemotherapy can elicit significant clinical responses in ovarian cancer patients who have undergone extensive pre-treatment (Holloway et al., 2023). A clinical trial enrolling patients with advanced ovarian cancer found that intraperitoneal administration of GL-ONC1 led to the prolongation of median progression-free survival (mPFS) from 4.5 months with standard treatment to 11 months (Zhang et al., 2009). GL-ONC1 (Olvi-Vec) is an oncolytic VV developed by Genelux in which the TK, hemagglutinin, and F145L genes in the viral genome were replaced with three specific expression cassettes (Sample and He, 2018), respectively encoding β-galactosidase, β-glucuronidase, and renin-luciferase/green fluorescent protein (RLuc-GFP) fusion proteins. This OV preparation has exhibited significant therapeutic efficacy in multiple cancers of the female reproductive system.

Melanoma and glioblastoma are among the primary indications for OV therapy. Melanoma arises from melanocytes located in the basal layer of the epidermis. Ultraviolet (UV) radiation is a major factor contributing to melanoma development through its harmful effects on the skin and its capacity to directly damage DNA (D’Orazio et al., 2013), thereby accelerating tumorigenesis. Intense and intermittent exposure to sunlight, as well as exposure to UV-A rays from artificial sources, are also associated with an increased risk of melanoma (Bevona et al., 2003). Approximately 25% of skin melanomas originate in preexisting moles (Replimune Group and Inc, 2024). Rigvir, launched in 2004, and Imlygic, launched in 2015, are both used to treat melanoma. In June 2024, Replimune announced pivotal results from the IGNYTE clinical trial, which investigated the combination of the investigational OV therapy RP1 with the PD-1 inhibitor nivolumab in melanoma patients resistant to prior anti-PD-1 therapy. According to the revised RECIST 1.1 criteria, the ORR was 33.6%, achieving the trial’s primary endpoint (Chmielowski et al., 2023; Kaufman et al., 2023). Furthermore, clinical trials evaluating RP2, either as monotherapy or in combination with nivolumab for choroidal melanoma treatment, showed that RP2 offered good safety and long-lasting anti-tumor activity in patients with metastatic choroidal melanoma when used alone or in combination with anti-PD-1 (Thomas et al., 2019). Replimune’s RPx platform currently includes RP1-RP3, which are based on a genetically engineered, selectively replicating HSV-1 backbone. These viruses contain insertions of codon-optimized human GM-CSF and a modified gibbon leukemia virus surface glycoprotein (GALV-GP R-) with the R sequence deleted, designed to maximize T cell activation and systemic immune stimulation (Khushalani et al., 2023; Wuhan Binhui Biotech Co Ltd., 2025). BS001 (OH2 injection) was developed by China Binhui Biopharmaceutical Co., Ltd. It is the world’s first oncolytic type II HSV (HSV-2) candidate drug to enter clinical research. This drug uses HSV-2 as a viral scaffold, removing the neurotoxic ICP34.5 gene and the immunosuppressive ICP47 gene from the viral genome while inserting the human GM-CSF gene, thereby endowing it with oncolytic activity and the ability to induce anti-tumor immune responses (Zhang et al., 2021). Clinical studies of BS001, both as monotherapy and in combination with PD-1 monoclonal antibodies, have yielded positive results across solid tumor types, including melanoma and colorectal cancer, demonstrating broad clinical application prospects (Tan et al., 2025; Author Anonymous, 2022). At present, BS001 is undergoing a critical phase III clinical trial for the treatment of melanoma in China (clinical trial registration number: ChiCTR2200061503) (Wang et al., 2023).

Glioma is a type of brain tumor that originates from glial cells and is one of the most common primary brain malignancies. In 2021, Japan granted the conditional approval of G47Δ for marketing in the treatment of malignant glioma, making it the first approved OV for primary brain tumors globally, and filling the gap in intracranial tumor immunotherapy (Boorjian et al., 2021). In 2023, Ad5-Ki67/IL-15, driven by the Ki67 promoter and loaded with IL-15, was developed and exhibited significant efficacy against glioblastoma *in vitro* (Zhang et al., 2020). In this construct, the promoter of the viral E1A gene, essential for early viral replication, was replaced with the Ki67 promoter, which is minimally expressed in normal brain tissue but highly expressed in glioblastoma multiforme (GBM) cells. Additionally, the human IL-15 gene was inserted into the E3 region under control of the CMV promoter, thereby amplifying both local oncolytic effects and systemic anti-tumor immune activity (Galanis et al., 2024). In 2024, researchers injected carcinoembryonic antigen (CEA)-expressing oncolytic measles virus derivative (MV-CEA) into patients with recurrent glioblastoma. Good treatment tolerance was observed, with no dose-limiting toxicity at the maximum feasible dose (2 × 10^0^ TCID50) (Peng et al., 2002). MV-CEA is a first-generation “traceable” oncolytic measles virus engineered by inserting the gene encoding the extracellular segment of human CEA into the genome of the attenuated Edmonston vaccine strain (MV-Edm) of measles virus (Msaouel et al., 2009). MV-Edm naturally targets tumor cells expressing high levels of CD46, conferring it natural tumor tropism. As CEA is secreted into the blood, viral replication levels can be monitored in real time through routine serum CEA testing during treatment, allowing for the non-invasive monitoring of drug efficacy (Göbel et al., 2023). The mechanism for enhancing OV specificity is multi-faceted, involving various approaches such as viral gene modification, response to the tumor microenvironment, immune system regulation, suicide gene system, combined treatment strategies, and targeted delivery technology, all of which are used in combination to maximize the enrichment and efficacy in tumor tissues while minimizing the impact on normal tissues (Romanishin et al., 2024). In addition, OV therapy has been applied to treat diseases colorectal cancer, ovarian cancer, liver cancer, and breast cancer.

## 5 Progress in the preparation of oncolytic viruses

### 5.1 The history of oncolytic virus technology development

The technological evolution of OVs has progressed through several critical stages: from the initial observation of viral effects on tumors to the systematic recording and analysis of such cases; from the identification and classification of effective viral strains to genetic engineering–based optimization; and from molecular biological characterization to large-scale production. These milestones have gradually provided robust support for the clinical application of OVs. The rapid advancement of OV technologies has been primarily driven by two core breakthroughs: (1) the identification and modification of viral strains, and (2) the optimization of manufacturing processes. These two developmental pathways continuously reinforce each other, forming the central driving engine for the entire field.

#### 5.1.1 Identification of viral strains

Candidate OV strains must possess the ability to selectively infect and kill tumor cells. The primary methods for identifying novel OVs include high-throughput screening and serial viral passaging (Todo et al., 2022b).

#### 5.1.2 Modification technologies

##### 5.1.2.1 Attenuation

The attenuation of OVs generally entails either deleting pathogenic viral genes or subjecting these pathogenic genes to tumor-specific regulation. In 1991, Martuza et al. reported the development of G207, an innovative OV product obtained by using genetic engineering technology to modify HSV-1, which was successfully applied in the treatment of glioma. Their modification approach entailed the deletion of both copies of ICP34.5 together with the inactivation of ICP6, which enabled G207 to replicate only within tumor cells without affecting normal cells (Ganly et al., 2000). Subsequent studies involved deletion of neurotoxic and immunosuppressive genes, such as ICP34.5 and ICP47 from HSV (Reid et al., 2002), AdV early genes E1A and E1B (Guse et al., 2011), and the thymidine kinase (TK) and viral growth factor (VGF) of VV (Hemminki et al., 2012).

As pathogenic viral genes are usually required for viral replication, their complete deletion will weaken the viral replication ability and lead to reduced oncolytic activity. To restrict this viral replication such that it only occurs within tumor tissues, researchers have employed tumor-specific regulatory mechanisms to control viral gene expression, such as tumor-specific promoter activation or tumor-specific miRNA deletion, thereby enhancing oncolytic activity while ensuring safety. The tumor-specific promoters employed for OV modification include the telomerase reverse transcriptase promoter (hTERT) (DeWeese et al., 2001), prostate-specific antigen promoter (PSA) (Jakubczak et al., 2003), alpha-fetoprotein promoter (AFP) (Jakubczak et al., 2003), E2F promoter (Ulasov et al., 2007), and Survivin promoter (Leber et al., 2018). OV modification strategies entailing the tumor-specific deletion of miRNAs have focused on transcripts including miR-124, miR-125, miR-143, and miR-145 (Duarte et al., 2012).

##### 5.1.2.2 Efficiency enhancement

Approaches ot enhancing the efficiency of OVs primarily entail the expression of suicide genes and the expression of molecules that stimulate the tumor immune microenvironment. Suicide genes are commonly used in gene therapy, and typically consist of prodrug-activating enzyme-encoding genes that convert non-toxic prodrugs into toxic compounds, thereby triggering cell death (Barton et al., 2006). By inserting these prodrug enzyme genes into the genome of OVs, the specific expression of the associated gene in tumor cells can be achieved, thereby generating significant local cytotoxic effects within tumors. The most commonly utilized prodrug enzyme systems are cytosine deaminase (CD), which converts the non-toxic fluorocytosine (5-FC) into the toxic fluorouracil (5-FU), and the phosphorylated form of herpes simplex thymidine kinase by ganciclovir (GCV) competes with GTP for incorporation during DNA replication, resulting in replication errors and cell cycle arrest (Pol et al., 2020; Foloppe et al., 2008; Mesnil et al., 1996).

A range of immunostimulatory gene-based modification strategies have been explored to date. The replication of OVs within tumor cells leads to cell lysis and the release of tumor antigens, thereby stimulating host immune recognition and attack. Through genetic engineering, OVs can carry and express specific immune-stimulating genes, further enhancing anti-tumor immune responses. These gene-encoded products include cytokines, chemokines, and other immune regulatory molecules that can improve immune system activity. Common immunostimulatory genes include GM-CSF, IL-12, IFN-γ, and the chemokines CXCL9 and CXCL10 (Fukuhara et al., 2016).

### 5.2 The process of preparing oncolytic viruses

The preparation of OVs is a complex and delicate process that involves multiple key steps, including cell culture, viral amplification, clarification, purification, ultrafiltration, and filling. After these stringent procedures, a high-quality viral product is obtained, characterized by precise tumor-targeting capacity and reliable safety.

Cell culture-based OV production has transitioned from traditional adherent (flat) culture systems to microcarrier-based or serum-free suspension cultures. These modern methods not only enable large-scale production but also reduce process-related impurities, establishing a solid foundation for the large-scale production of OVs (Shen et al., 2024; Göbel et al., 2024; Morenweiser, 2005). In addition, perfusion culture methods can facilitate the continuous replenishment of fresh culture medium while maintaining an optimal cell growth environment and improving nutrient utilization rates. During viral amplification, perfusion enables continuous harvesting of virus-containing supernatant, thereby increasing overall viral yield. Cell growth in serum-free medium can completely remove animal-derived components, providing the highest guarantee of the safety of commercial products. However, the absence of a fully optimized commercial serum-free medium tailored for OV production remains a major technical challenge.

During the virus harvesting stage, the conventional freeze–thaw method, although effective in lysing cells, is limited by its requirement for extensive cold-chain infrastructure and large storage capacity, which restricts production scalability. As a result, continuous-flow treatment technologies based on mechanical force or chemical action have gradually replaced freeze-thaw techniques as the most common virus harvesting strategy. Among these, tangential flow filtration and homogenization treatment are the two leading approaches (Loewe et al., 2020).

With respect to clarification and purification, the early-stage removal of impurities has traditionally relied on centrifugation, but its utility for large-scale production is limited by its high energy consumption and limited processing scale. The clarification and filtration process has thus emerged as a prominent alternative to centrifugation, while column chromatography purification has replaced density gradient centrifugation in the context of virus purification, affording higher purification efficiency and virus recovery (Kearney, 2023). Moreover, among chromatographic techniques, membrane chromatography and monolithic column chromatography offer distinct advantages over traditional packed-bed columns, including shorter processing times, higher loading capacities, and larger pore sizes. These features significantly enhance purification efficiency and scalability for OV manufacturing (Loewe et al., 2022).

In terms of ultrafiltration and buffer replacement, membrane-based approaches and hollow fiber ultrafiltration have gradually replaced ultracentrifugation owing to their greater efficiency, making them ideal for virus concentration and buffer replacement. This transformation not only improves production efficiency, but also reduces production costs, providing strong support for the large-scale production and clinical application of OVs (U.S. Food and Drug Administration, 2024).

With respect to virus preparation strategies, OV formulations are usually classified into liquid and freeze-dried preparations. Compared with liquid preparations, freeze-dried preparations exhibit greater stability and less restrictive cold-chain requirements. Owing to its complex preparation process and stringent requirements for excipient formulations and freeze-drying equipment, viral activity may be reduced. At present, with the exception of certain strains (such as the oncolytic virus M1), injectable aqueous formulations are most commonly used in clinical practice, as challenges related to lyophilization remain unresolved. Moreover, the three-dimensional structural characteristics of virus particles to some extent determine whether they are suitable for development as freeze-dried preparations.

In terms of virus quality control, OV product lines cover a wide range of viruses, exhibiting various differences in biological behavior, mechanisms of action, and genetic modification strategies. Therefore, it is necessary to tailor corresponding production processes and quality control measures for the unique risk spectrum of each virus. The quality control framework for OV products involves multiple analytical dimensions, including but not limited to the determination of exogenous factors, PCR identification, NGS sequencing, viral titer measurement, biological activity detection, cytopathic effect determination, chromatographic analysis, protein identification, and genetic modification verification (Harrington et al., 2019).

## 6 Challenges and trends in oncolytic drug development

After years of development, some OV drugs have gained market recognition, and many more show promising prospects in clinical trials. With continuous updates and iterations in cell and gene therapy technologies, the development of production and purification processes, the expansion of clinical indications, and the improvement of combination therapy strategies, OVs offer significant potential and broad application prospects. However, several challenges persist, including the specific risks associated with patients with compromised immune function,the limited efficacy and resistance of monotherapy, difficulties in achieving systemic delivery, the need for precision therapy, and constraints related to large-scale manufacturing (Yoon et al., 2020). In the coming years, these challenges are anticipated to drive the emergence of new development trends, supported by innovative technologies and methodologies.

Although OV has been engineered to have certain tumor selectivity, its safety considerations for clinical application, especially in patients with particularly low immune function (such as those undergoing high-intensity chemotherapy, after hematopoietic stem cell transplantation, or those with primary immunodeficiency), still require extreme caution. In these patients, the “immune firewall” function of the body to clear and control viruses is severely impaired, which may lead to uncontrolled viral replication. The “selective” replication of OV in tumor cells is relative. In an extremely immunodeficient environment, even if the replication efficiency of the virus in healthy cells is low, it may continue to accumulate due to the lack of immune surveillance, thereby posing a risk of infection in healthy tissues, such as disseminated viral sepsis or organ-specific inflammation (such as hepatitis, pneumonia) (Lemos de Matos et al., 2020). Furthermore, the massive replication of viruses or the immune-stimulating factors they carry (such as GM-CSF) may also trigger life-threatening cytokine release syndrome (CRS) (Andtbacka et al., 2015). Therefore, for clinical trials targeting this patient group, strict risk stratification is necessary. Typically, patients with severe immunodeficiency are excluded, and rigorous virological monitoring and emergency antiviral protocols are employed. This also reminds us that the optimal distant anti-tumor immune effect of OV actually depends on a basically functional immune system, rather than a completely immunosuppressed state.

There are still several practical challenges limiting OV application, including limited monotherapy efficacy and drug resistance, but several promising technologies have the potential to overcome these issues. The CRISPR/Cas9 system, as an efficient and precise gene editing tool, holds great promise for OV development. Researchers can make targeted modifications to OVs to significantly enhance their specificity and efficiency when recognizing and killing tumor cells. For instance, researchers have employed CRISPR/Cas9 to modify viral genomes, enabling selective targeting of cancer cells deficient in EGFR or RAS, which demonstrated notable anti-tumor effects (Phelps et al., 2018; Cai et al., 2020). CRISPR/Cas9 can also be used to delete or insert specific genes, thereby enhancing the lethality and persistence of OVs (Ebrahimi et al., 2020; Lovatt et al., 2023).

The anti-tumor efficacy of OVs can also be enhanced through combination therapy, such as the combination with ICIs. Some trials have successfully evaluated the safety and efficacy of combining OVs with PD-1 and CTLA-4 inhibitors, offering a strong foundation for their clinical application (Macedo et al., 2020). Combining OVs and chemotherapeutic drugs has shown promising efficacy in various cancers. For example, in clinical trials focused on melanoma and gastrointestinal cancer, combination therapy significantly improved OS and PFS in patients (Kochneva et al., 2020). The combination of OVs and molecularly targeted drugs also represents a promising treatment approach with the potential to enhance anti-tumor efficacy and overcome drug resistance. Similarly, the combination of OVs with immunotherapeutic agents is also a promising approach to cancer treatment. For example, while CAR-T cell therapy has shown remarkable efficacy in hematologic malignancies, it faces challenges in solid tumors due to the immunosuppressive tumor microenvironment and heterogeneous antigen expression. OVs can enhance CAR-T cell infiltration by altering the composition of the tumor microenvironment and can be engineered to express cytokines to enhance CAR-T cell function (Kan et al., 2021). OVs can also be used in combination with radiotherapy. Because radiotherapy can increase the permeability of tumor cell membranes, this combination can improve OV infection rates and afford more efficient viral replication. By modulating the expression of specific genes of interest, developing novel OVs can also achieve outcomes such as enhanced oncolytic activity, immune cell activation, the induction of specific immune responses, improved tumor microenvironment composition, the alleviation of immune suppression, and the regulation of tumor metabolism.

Reverse genetics provides an efficient platform for the targeted modification of RNA viral genomes. This technology can reverse-transcribe viral RNA into complementary DNA (cDNA) and clone it into a manipulable plasmid vector, thus achieving precision editing at the DNA level. Live viruses can then be generated via *in vitro* transcription (IVT) or the direct transfection of recombinant cDNA into cells. This technology is widely applied in viral research, vaccine development, and gene therapy, providing technical support for personalized design and precise engineering of OVs (Kennedy et al., 2022). The establishment of oncolytic nanoparticles (ONPs) offers another approach to OV delivery and release. This strategy entails the initial acquisition of the viral genome in the form of DNA or RNA using bacterial artificial chromosomes (BAC), high-copy plasmids, PCR amplification, and IVT, after which it can be encapsulated within liposomes to form nanoscale ONPs. These nanoparticles are capable of efficiently penetrating gaps in tumor tissues and can be readily taken up by tumor cells, after which the viral nucleic acids are released, initiating the assembly of live replicating virions within cells, resulting in cascade diffusion and the oncolytic effects of viral progeny (Heo et al., 2013). This strategy greatly enhances OV targeting and bioavailability while enabling precise delivery and controlled release.

Currently, most marketed or clinically investigated OVs are administered via intratumoral injection, which can offer certain advantages for the treatment of single lesions or limited numbers of metastatic lesions. However, for deep tumors and multiple metastatic lesions, intratumoral injection is often infeasible and hampered by a combination of procedural complexity and high operational risk. Therefore, a systematic drug delivery method needs to be developed to overcome these issues. However, to achieve effective systemic delivery of oncolytic viruses, several key obstacles they face in the bloodstream must be overcome: including rapid dilution, non-specific capture by blood cells, and rapid neutralization and clearance by the host’s innate and adaptive immune systems. To address these challenges, current research involves, on the one hand, using high-throughput screening platforms to search for new oncolytic viruses with lower natural infectivity and controllable immunogenicity; on the other hand, modifying the surface structure of the viruses through genetic and chemical engineering methods, aiming to enhance their retention time in the blood and endow them with the ability to actively target tumors. At the same time, developing delivery systems based on nanoparticles or living cells, providing “invisibility” protection for the viruses, has become an important way to reduce their loss during delivery.While intratumoral injection remains the most common approach, several OVs capable of systemic administration have already entered clinical trials, including oncolytic VV JX-594 (Ruano et al., 2020), oncolytic VV KM1 (Andtbacka et al., 2015), and oncolytic hepatitis A virus M1 (Song et al., 2022).

In advanced-stage disease, tumors develop rapidly, leaving a short treatment window. As such, there is an urgent need for the identification of predictive biomarkers associated with the efficacy of approved and investigational therapies. Wuhan Binhui Biotechnology Co., Ltd. is currently exploring biomarkers associated with OH2 tumor treatment outcomes. In the case of the M1 virus, MXRA8 and ZAP have been identified as dual biomarkers predictive of therapeutic efficacy (Predictive: Used for screening patients who may benefit), offering valuable guidance for patient selection in clinical trials (Wang et al., 2024).

When manufacturing OVs, it is particularly important to remove animal-derived components due to their potential safety risks and heterogeneity across different batches. Therefore, establishing serum-free culture processes and searching for “animal-free” alternative materials have emerged as key directions for OV manufacturing efforts (U.S. Food and Drug Administration, 2020). At the same time, it is essential to maintain high viral activity and stability, which are critical determinants of therapeutic efficacy. Special attention must be paid to preventing the inactivation of the virus and to maintaining drug stability during production, storage, transportation, and clinical application. The freeze-drying process can significantly improve the stability of OVs while reducing the dependence on cold-chain logistics, thus improving the accessibility and convenience of these drugs in different environments. OV purification technologies are expected to evolve toward greater scale, automation, and intelligence in the future. With rising market demand, large-scale, automated, and smart manufacturing will become essential for improving production efficiency, reducing manual intervention, and ensuring consistent product quality.

## 7 Quality control strategies for oncolytic viruses

As advanced biological therapeutic products, OVs require rigorous quality control and regulatory oversight by appropriate agencies throughout the globe. Because OVs are live, replicating biological products, regulating their quality is far more complex than for traditional drugs, requiring the coverage of the entire chain of production—from virus seed banks to the release of the final products. The FDA classifies OVs as “gene therapy products” within the category of “biological products” (Heo et al., 2013). EMA classifies OVs as “gene therapy drugs” under the “advanced therapeutic drugs” category, and imposes a regulatory framework that is more centralized and strict (European Parliament and Council of the European Union, 2007). The FDA emphasizes product consistency, safety, and efficacy, providing support for clinical trials. In addition to safety and effectiveness, EMA places great attention on environmental risks, viral shedding, and genetic stability, providing a stronger overall risk management perspective. Following the establishment of these regulatory frameworks by the FDA and EMA, China has recently taken an important step toward standardizing their advanced therapy medicine guidelines. On 10 June 2025, the Center for Drug Evaluation (CDE) of the China National Medical Products Administration released the “Scope, Classification, and Interpretation of Advanced therapy medicinal products (Exposure Draft)”, which proposes a clear definition and three major classifications for advanced therapeutic medicinal products (ATMPs) in China, including cell therapies, gene therapies, and other innovative products. These guidelines classify OVs as “gene therapy drugs” under the subclassification of “oncolytic microbial drugs”, which include OVs and oncolytic bacteria (Center for Drug Evaluation and National Medical Products Administration, 2025). The CDE has incorporated OVs into the regulatory framework for gene therapy products, providing a clear direction for their quality control. The quality control of OVs must therefore strictly adhere to the relevant principles and technical requirements governing gene therapy products, with particular emphasis on their virological characteristics and genetic stability. Although the FDA, EMA, and CDE each maintain distinct regulatory frameworks with differing focal points, they share several core quality control modules (Table 3).

**TABLE 3:** Core quality control modules for OVs.

Module	Critical control point
Virus seed/cell bank	Origin, passage history, gene sequence, replication ability, non-exogenous factors
Starting raw materials	Plasmids, serum, trypsin, nucleases, packaging cells
Process control	Upstream and downstream key steps, virus harvesting, purification, inactivation/sterilization, filling
Quality attributes and release standards	Identification, potency, purity, impurities, and safety-related
Stability	Temperature, light, freeze-thaw, aging
Biosafety/environment	The sealing grade of the factory building, inactivation of waste liquid, and immunization of operators

This quality control concept, which is in line with that of FDA/EMA, is based on risk assessment and runs throughout the entire life cycle, has also been deeply integrated and reflected in China’s regulatory practice for oncolytic viruses. On 13 February 2023, the CDE issued the “Technical Guidelines for Pharmaceutical Research and Evaluation of OVs Products (Trial)”, which provides a comprehensive, life-cycle quality control framework for OV products. The guideline emphasizes controlling exogenous factors, genetic stability, and biological activity based on the intrinsic risk characteristics of replicable viruses. It also recommends adopting the “Critical Quality Attribute (CQA) + Critical Process Parameter (CPP)” model to formulate release standards (Center for Drug Evaluation and National Medical Products Administration, 2023). The publication of this guideline highlights the new standards for OV standardization and normalization in China, providing an authoritative technical foundation for the research and production of OVs in the country.

## 8 Conclusion and future perspectives

The OV field has moved from the initial observation of wild strains to a systems-focused era of precise engineering and multi-modal collaboration. Over the past 2 decades, OV products have been launched successfully, and more than 200 clinical trials have been initiated, underscoring the clinical feasibility and industrial utility of these viral therapeutics. However, the design and construction of new OVs to improve their anti-tumor selectivity and efficacy will require extensive ongoing preclinical and clinical research. Through their genomic plasticity and continuous advances in genetic engineering methods, OVs have been effectively leveraged for precise targeting, payload diversification, and synergistic lethality. When combined with other therapeutic approaches including chemotherapy, ICIs, and CAR-T cells, OVs can reshape the tumor immune microenvironment and interfere with drug resistance. OVs are thus viewed as a promising synergistic platform for overcoming acquired therapeutic resistance and immune tolerance in solid tumors, while also offering opportunities for overcoming tumor heterogeneity and recurrence. To fully unleash the clinical potential of OVs, however, there remains an urgent need to systematically analyze the mechanisms governing their immunobiological activity. Subsequent research should focus on precision viral skeleton engineering, the evidence-based optimization of combined strategies, and the three-dimensional evaluation of delivery pathways. Only through continuous bidirectional validation through basic to clinical research can this treatment paradigm be advanced further, ultimately extending the benefits of OVs to a wide range of solid tumors.

Meanwhile, as oncolytic virus therapy progresses from clinical research to wider application, its long-term ecological safety has become an issue that requires forward-looking assessment. From an ecological perspective, the large-scale use of genetically engineered replicative virus preparations theoretically poses potential risks such as virus environmental release, genetic recombination with wild-type viruses, and unexpected impacts on non-target organisms. However, existing OV designs (such as multiple attenuation and tumor-dependent replication) and strict regulatory frameworks (such as review requirements for virus shedding and environmental risks) have provided a solid foundation for controlling these risks. In the future, ensuring the sustainable development of this field not only depends on the continuous improvement of therapeutic efficacy, but also lies in establishing an ecological risk assessment and active monitoring system covering the entire life cycle. This includes developing next-generation viruses with lower environmental stability, improving medical waste disposal regulations, and conducting long-term monitoring of virus sequences in environmental media such as wastewater. Only by coordinating the advancement of efficacy, safety, and ecological responsibility can this breakthrough technology in oncolytic viruses truly achieve its long-term value of benefiting humanity and become a reliable and sustainable choice in the arsenal of tumor treatment.
